# Circular RNA has_circ_0067934 is upregulated in esophageal squamous cell carcinoma and promoted proliferation

**DOI:** 10.1038/srep35576

**Published:** 2016-10-18

**Authors:** Wenjia Xia, Mantang Qiu, Rui Chen, Siwei Wang, Xuechun Leng, Jie Wang, Youtao Xu, Jingwen Hu, Gaochao Dong, Prof Lin Xu, Rong Yin

**Affiliations:** 1 Department of Thoracic Surgery, Nanjing Medical University Affiliated Cancer Hospital, Jiangsu Key Laboratory of Molecular and Translational Cancer Research, Cancer Institute of Jiangsu Province, Nanjing 210009, China; 2 The Fourth Clinical College of Nanjing Medical University, Nanjing, 210000, China; 3 Department of Cardiothoracic Surgery, Taixing People’s Hospital, The Affiliated Taixing Hospital of Yangzhou University, Taixing 225400, China; 4 Department of Thoracic Surgery, Huai’an First People’s Hospital, Nanjing Medical University, Huai’an 223300, China; 5 Department of Scientific Research, Nanjing Medical University Affiliated Cancer Hospital, Cancer Institute of Jiangsu Province, Nanjing 210009, China

## Abstract

Esophageal squamous cell carcinoma (ESCC) is one of the most prevalent and deadly types of cancer worldwide especially in Eastern Asia and the prognosis of ESCC remain poor. Recent evidence suggests that circular RNAs (circRNAs) play important roles in multiple diseases, including cancer. In this study, we characterized a novel circRNA termed hsa_circ_0067934 in ESCC tumor tissues and cell lines. We analyzed a cohort of 51 patients and found that hsa_circ_0067934 was significantly overexpressed in ESCC tissues compared with paired adjacent normal tissues. The high expression level of hsa_circ_0067934 was associated with poor differentiation (*P* = 0.025), I-II T stage (*P* = *0.04*), and I-II TNM stage (*P* = *0.021*). The *in vitro* silence of hsa_circ_0067934 by siRNA inhibited the proliferation and migration of ESCC cells and blocked cell cycle progression. Cell fraction analyses and fluorescence *in situ* hybridization detected that hsa_circ_0067934 was mostly located in the cytoplasm. Our findings suggest that hsa_circ_0067934 is upregulated in ESCC tumor tissue. Our data suggest that hsa_circ_0067934 represents a novel potential biomarker and therapeutic target of ESCC.

Esophageal cancer is the eighth most common cancer and the sixth leading cause of cancer death worldwide^1^,^2^. One of the major disease subtypes is esophageal squamous cell carcinoma (ESCC), which is a tumor arising from esophageal epithelial cells^3^. The overall 5-year survival rate of stage III ESCC patients is only 10–15%, and the median survival of stage IV patients is less than 1 year. Cancer is widely regarded as a genetic disease, and ESCC is no exception. However, the molecular and genetic basis of esophageal carcinogenesis has not been clearly elucidated. The prognosis of ESCC remains poor due to an incomplete understanding of the molecular mechanisms of ESCC development and progression^4^,^5^,^6^. Therefore, it is critical to identify new biomarkers and therapeutic targets to improve ESCC diagnosis and treatment.

Although circular RNA was first reported more than 20 years ago, these molecules were considered byproducts of splicing errors^7^. The recent development of high-throughput RNA sequencing (RNA-Seq) has led to the direct detection of more circular RNAs in eukaryotic cells. Circular RNAs are novel RNA molecules that are formed by back-splicing covalently joined 3′- and 5′-ends. These molecules generally do not encode protein. However, these RNAs can occur in any genomic region, and 85% of circular RNAs are aligned in the sense orientation with known protein-coding genes and can span 1–5 exons^8^,^9^,^10^,^11^,^12^,^13^,^14^,^15^. Many circular RNAs have been identified in recent studies. One example is SRY, which is a testis determining gene^10^. The best known circular RNA is CDR1, which encodes cerebellar degeneration-related protein 1. This RNA has been shown to bind miRNA-7^16^. Circular RNAs are considered extremely rare in nature^7^. Recent data suggest circular RNAs could sponge miRNAs and are enriched with functional miRNA binding sites^16^,^17^. Mature miRNAs have important regulatory roles in cell growth, proliferation, differentiation, and cell death^18^. However, whether circular RNAs harbor miRNAs with regulatory roles in ESCC is still unknown.

We have examined the expression profile of circRNAs in non-small cell lung cancer with microarray and found the circRNA hsa_circ_0067934 was significantly overexpressed. The circRNA hsa_circ_0067934 is generated from chromosomal region 3q26.2, which is back spliced by exon 15 and exon 16 of PRKCI. Mature has_circ_0067934 transcript is a circular RNA molecule of 170 nt. According to the circBase database, has_circ_0067934 can be detected in many types of cancer cell lines^19^, like K562 and A549.

(http://www.circbase.org/cgi-bin/singlerecord.cgi?id=hsa_circ_0067934).

However, the relationship between the expression level of hsa_circ_0067934 and ESCC is unclear. Therefore, in this study we characterized expression of hsa_circ_0067934 in ESCC and investigated its biological function *in vitro*.

## Results

### Characterization of hsa_circ_0067934 in ESCC cells and tissues

**Figure 1:**
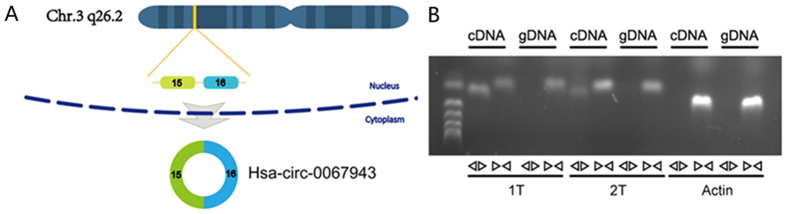
Characterization of hsa_circ_0067934 in ESCC cells and tissue. (**A**) Two exons form hsa_circ_0067934 by back splicing from chromosomal region 3q26.2. (**B**) Divergent primers detect circular RNAs in cDNA but not genomic DNA (gDNA).

The circRNA hsa_circ_0067934 is generated from chromosomal region 3q26.2, which contains two exons that create hsa_circ_0067934 by back splicing (Fig. 1A). We used RT-PCR assays to verify the expression of hsa_circ_0067934. We then designed two sets of primers for hsa_circ_0067934. The first set contained a divergent primer that amplifies only the circular form. The second set of primers contained an opposite-directed primer to detect the linear forms. The schematic diagram of primers was shown in supplementary files. PCR results indicate the circular form was amplified using the divergent primers (Fig 1B). PCR Assays using cDNA and genomic DNA as templates did not produce amplifications with the divergent primers. Actin was used as a linear RNA control (Fig. 1B). These results demonstrate hsa_circ_0067934 is consistently expressed in ESCC tumor tissues.

### Correlation between hsa_circ_0067934 expression and clinical characteristics

**Figure 2:**
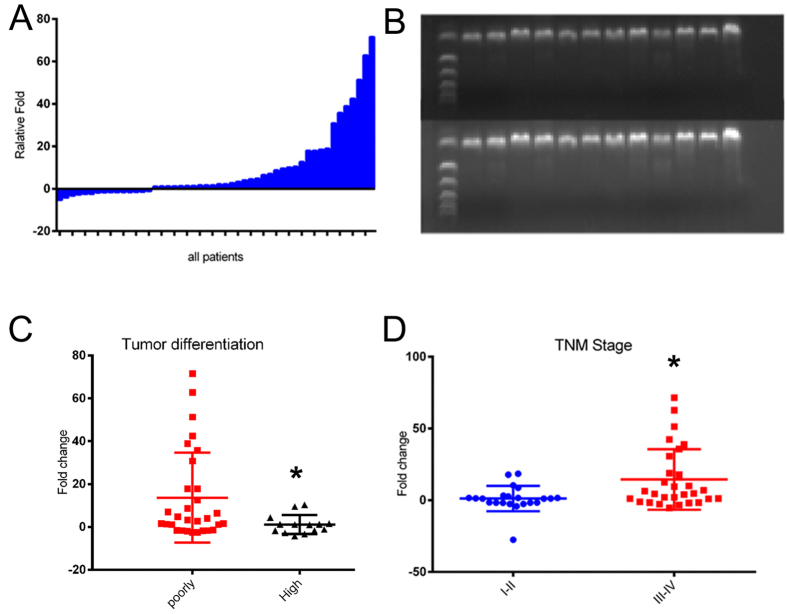
Analysis of hsa_circ_0067934 expression in ESCC tissues and clinical parameters. (**A**) hsa_circ_0067934 was detected in 51 pairs of ESCC tissues by qRT-PCR. The levels of hsa_circ_0067934 in ESCC tissues are significantly higher than those in non-tumor tissues (*P* = 0.011). Nucleic acid electrophoresis directly shows that hsa_circ_0067934 expression was upregulation in ESCC tumor tissues (The image below means the ESCC tumor tissue). Hsa_circ_0067934 was upregulated inpatients with poor differentiation and TNM III-IV stage. *P < 0.05. Error bars indicate S.E.M.

The expression of hsa_circ_0067934 was detected in 51 pairs of primary cancerous and adjacent noncancerous tissues derived from ESCC patients using qRT-PCR. We found that hsa_circ_0067934 was significantly overexpressed in ESCC, and the average upregulation fold was 8.75 (p = 0.011) (Fig. 2A). We also evaluated the PCR product by nucleic acid electrophoresis to confirm the upregulation of hsa_circ_0067934 in ESCC tumor tissue (B). We next evaluated the association between hsa_circ_0067934 and clinic pathological parameters (Table 1). As shown in Table 1, tumor differentiation, T stage, and TNM stage (*P* < 0.05) were significantly associated with the expression level of hsa_circ_0067934. We found patients with poorly differentiated tumors had higher hsa_circ_0067934 expression level (Fig. 2C) (poorly vs. high: 28.40 vs. 17.64). The patients with TNM I-II showed higher hsa_circ_0067934 expression than patients with TNM III-IV (Fig. 2D, TNM I-II vs. TNM III-IV: 20.48 vs. 30.19, *P* = 0.021 < 0.001). The expression of hsa_circ_0067934 was not correlated with other clinical factors such as age, cancer location, lymph node metastasis, or tumor size, etc. The expression of PRKCI was also analyzed among 26 patients, we found the expression between PRKCI and has_circ_0067934 were not significantly correlated (supplementary file, *P* = 0.136). In conclusion, hsa_circ_0067934 expression was upregulated in ESCC tumor tissue and is correlated with TNM stages.

### Expression of hsa_circ_0067934 in ESCC cell lines

**Figure 3:**
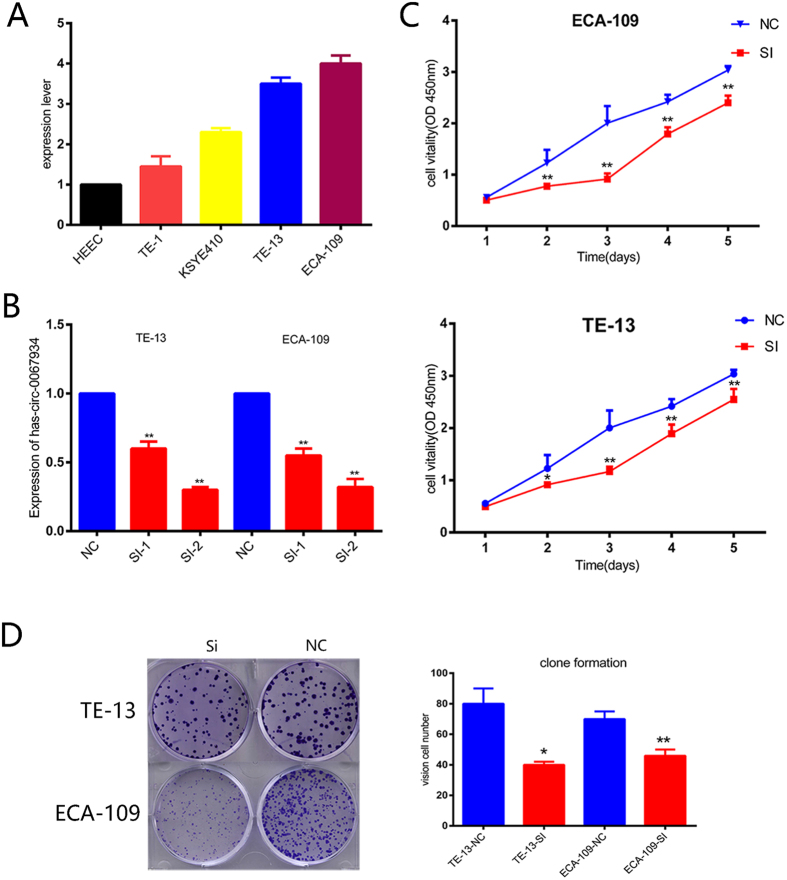
Expression of hsa_circ_0067934 in ESCC cell lines shows hsa_circ_0067934 promotes proliferation of ESCC cell lines *in vitro*. (**A**) hsa_circ_0067934 was upregulated among ESCC cells including TE-13 and ECA-109. (**B**) The inhibitor si2-hsa_circ_0067934 shows better inhibition efficiency. (**C**) CCK-8 kit shows that si-hsa_circ_0067934 inhibited cell proliferation in TE-13 cells and ECA-109 cells. (**D**) Clone formation shows the same result as the CCK8 kit. F: *P < 0.05, **P < 0.01. Error bars indicate S.E.M.

**Figure 4:**
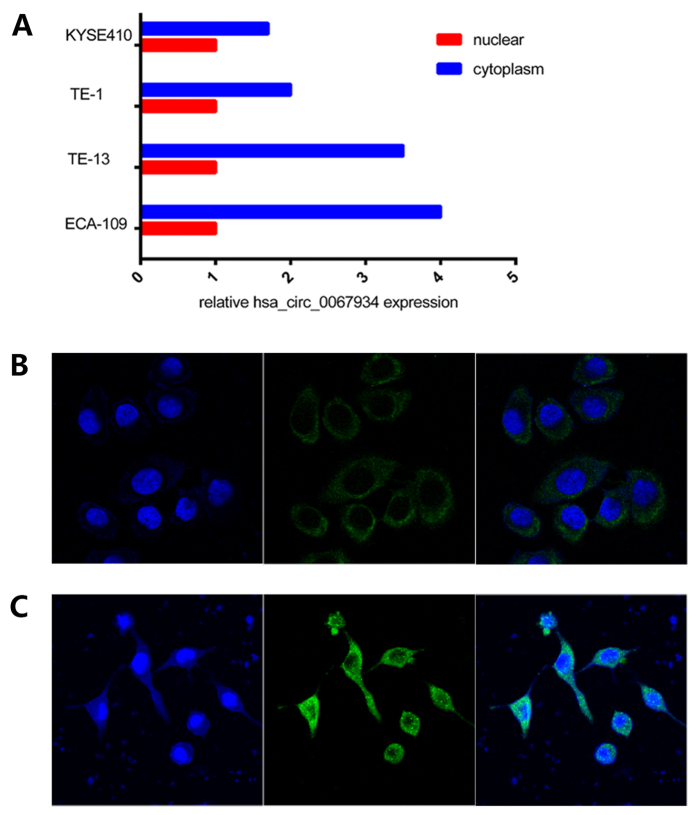
Identify the localization of hsa_circ_0067934 in ESCC cells. (**A**) The majority of hsa_circ_0067934 was located in the cytoplasm through the nuclear mass separation experiment; FISH assay also shown has_circ_0067934 was mostly located in cytoplasm of ECA109 (**B**) and TE-13 cells (**C**).

We first examined the expression of hsa_circ_0067934 in esophageal cancer cell lines by qRT-PCR before investigating its function *in vitro*. The expression level of hsa_circ_0067934 was upregulated in all analyzed ESCC cells when normalized to HEEC (Fig. 3A). We found hsa_circ_0067934 was most upregulated in TE-13 and ECA-109 cells. Therefore, TE-13 and ECA-109 were selected as our experimental cell lines. We determined the subcellular localization of hsa_circ_0067934 in different ESCC cell lines by the nuclear mass separation assay. As shown, the majority of hsa_circ_0067934 was present in the cytoplasm (Fig. 4A). FISH assay was performed to characterize has_circ_0067934 expression visually. And as shown, has_circ_0067934 was mostly located in cytoplasm (Fig. 4B,C). Our results show that hsa_circ_0067934 is significantly upregulated in TE-13 and ECA-109 cells, and mostly locates in cytoplasm.

### The circRNA hsa_circ_0067934 promotes proliferation and migration of ESCC cell lines *in vitro*

**Figure 5:**
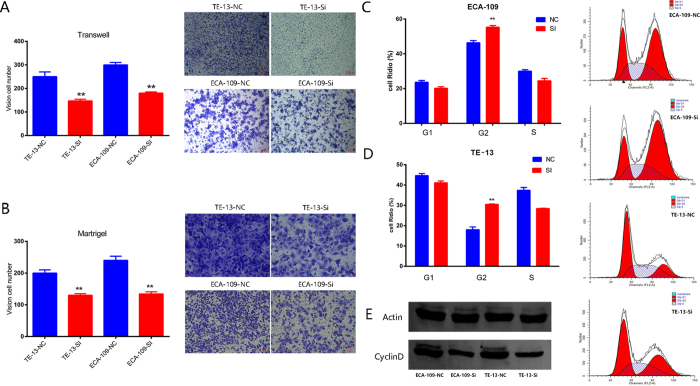
Hsa_circ_0067934 promotes proliferation of ESCC cell lines *in vitro* and alters the cell cycle of ESCC cell lines. (**A**) After transfecting si-hsa_circ_0067934, ESCC cells showed reduced migration in transwell migration assays. (**B**) hsa_circ_0067934 also influenced the invasion ability of TE-13 and ECA-109 cells. (**C,D**) ECA-109 and TE-13 cells transfected with si- hsa_circ_0067934 were blocked at the G2 phase. (**E**) The protein levels of cyclin D1 were downgraded in cells transfected with si-hsa_circ_0067934. F: *P < 0.05, **P < 0.01. Error bars indicate S.E.M.G: All the gels in this article have been run under the same experimental conditions.

We designed two siRNAs to inhibit hsa_circ_0067934 and test its biological function *in vitro*. qRT-PCR showed that si2-hsa_circ_0067934 had superior inhibition efficacy and si2-hsa_circ_0067934 was used in further functional experiments (Fig. 3B). The cell lines TE-13 and ECA-109 cells were transfected with si- hsa_circ_0067934 or negative control (NC). 36 h after treatment, the expression levels of hsa_circ_0067934 were effectively decreased (Fig. 3B). The CCK8 assay results showed that knockdown of hsa_circ_0067934 significantly inhibited the proliferation of both ECA-109 and TE-13 cell lines (Fig. 3C). Furthermore, the clone formation experiment also showed that si-hsa_circ_0067934 inhibited cell proliferation (Fig. 3D). We then performed flow cytometric analyses to further evaluate whether hsa_circ_0067934 affects proliferation of ESCC cells by altering apoptosis or cell cycle progression. TE-13 and ECA-109 cells transfected with si-hsa_circ_0067934 were arrested at the G2 phase (Fig. 5C,D). However, hsa_circ_0067934 did not affect apoptosis (supplementary file). In order to verify the cause of cell cycle change, the cyclin D protein levels were assessed in ESCC cells (Eca-109 cells and TE-13 cells) by western blot after si-hsa_circ_0067934 or si-NC transfection (Fig. 5E). The results of the transwell assay showed that siRNA treatment significantly impaired the migration capacity compared to NC (Fig. 5A). The inhibition of hsa_circ_0067934 also inhibited the invasion ability of TE-13 and ECA-109 cells (Fig. 5B). Our results suggest that hsa_circ_0067934 promotes the motility and migration of ESCC cells and affects cell cycle status.

## Discussion

The majority of mature messenger RNAs are linear molecules with 5′ and 3′ termini that reflect the start and stop of RNA polymerase on the DNA template^20^. Various RNA molecules can be combined by splicing reactions (trans-splicing) in cells. However, the covalent linkage of the ends of a single RNA molecule to form a circular RNA (circRNA) is a rare event^7^,^8^,^10^. CircRNAs were first discovered in plants and were shown to encode subviral agents thirty years ago^7^. Many mammalian genes have been shown to generate circular RNA isoforms at low levels by microscopy. For example, both MLL and ETS-1 form circRNAs^21^,^22^. There are well known circRNAs (SRY and CDR1AS) that harbor miRNAs with a regulatory function^10^,^16^. The circular RNA FOXO3 forms ternary complexes with p21 and CDK2 to retard cell cycle progression^23^. The cumulative evidence shows that circRNAs play an important role in disease development.

Cancer is a complex dynamic biological processes, and esophageal cancer is no exception^24^. Multiple genes are associated with ESCC. Additionally, the lncRNAs HOTAIR and HNF1A-AS^25^,^26^ and microRNAs such as microRNA-98 and microRNA-214 are also associated with ESCC^18^. It is not currently clear in the literature whether circRNAs are associated with ESCC. There is currently one publication suggesting that circRNAs are related to ESCC^17^. In this study, we identified a new circRNA hsa_circ_0067934 and hypothesized that it is related to ESCC. We also reported that this circRNA is associated with esophageal cancer development and demonstrated that it has an important role in esophageal cancer.

In this study, we demonstrated the expression of hsa_circ_0067934 in ESCC and assessed the correlation with clinical factors. We then explored the potential function of hsa_circ_0067934 by siRNA mediated silencing.

Our data indicate hsa_circ_0067934 was significantly upregulated in ESCC tumor tissue. We also found the upregulation of hsa_circ_0067934 was related to tumor differentiation, T stage, and TNM stage. It is well accepted that poorly differentiated and moderately differentiated carcinomas have worse prognoses than high differentiation carcinoma^27^. TNM staging is a classic evaluation of prognostic criteria, and the patients with stage III and IV have a worse prognosis than patients with TNM I and II^28^. In summary, hsa_circ_0067934 may represent a potential biomarker for assessing the risk of esophageal cancer and could function as a marker of ESCC prognosis.

We found that hsa_circ_0067934 promoted the proliferation and migration of ESCC cell lines *in vitro*. We also found siRNA treated ESCC cells had blocked cell cycle in G2 phase by flow cytometric analysis. Therefore, we hypothesize that hsa_circ_0067934 promotes the proliferation of ECSS cells by regulating the cell cycle. In consistence with previous reports, the expression of has_circ_0067934 is not correlated with its host gene, PRKCI. We found that circRNAs were located in the cytoplasm, which is consistent with previous studies^10^,^16^,^29^. Our results also show that the majority of hsa_circ_0067934 was present in the cytoplasm, which indicates that the underlying molecular mechanism of hsa_circ_0067934 is possible post-transcriptional regulation. The specific mechanisms include binding to miRNAs to regulate the expression of mRNA and the formation of complexes with cell cycle proteins. And we will investigate the molecular mechanism of hsa_circ_0067934 in future studies.

In summary, hsa_circ_0067934 is upregulated in ESCC tumor tissues, and its expression is related to tumor differentiation, T stage, and TNM stage. The reduction of hsa_circ_0067934 inhibits the proliferation and migration of ESCC cells.

## Material and Methods

### Patients and tissue samples

All primary ESCC tissues and adjacent normal tissues were collected from patients who had undergone surgery at the Department of Thoracic Surgery, Cancer Institute of Jiangsu Province, between 2013 and 2014. All tumors and paired normal tissues were confirmed by experienced pathologists. The clinical and pathological characteristics for each patient were collected after surgery. We obtained written informed consent from all patients. The study was approved by the Ethics Committee of the Cancer Institute of Jiangsu Province. The study was in accordance with the provisions of Ethics Committee of Nanjing Medical University. I confirmed that all methods were performed in accordance with the relevant guidelines and regulations.

### RNA extraction and qRT-PCR analyses

The RNA was extracted from tissues or cultured cells with Trizol reagent according to the manufacturer’s protocol (Life Technologies, Scotland, UK). Then, 500 ng of total RNA was reverse transcribed in a final volume of 10 μl using random primers and standard conditions with the Prime Script RT Master Mix (Takara, Cat. #RR036A). Then, we performed quantitative real-time polymerase chain reaction (qRT-PCR) using the SYBR Select Master Mix (Applied Biosystems, cat:4472908) with 0.5 μl complementary DNA (cDNA) on the ABI7300 system (Applied Biosystems, Foster City, CA, USA) according to the manufacturer’s instructions. We used GAPDH, β-actin, and snRNA U6 as internal controls and tested hsa_circ_0067934 and PRKCI expression levels by qRT-PCR. We used the following primer sequences: hsa_circ_0067934:forward, 5′-TAGCAGTTCCCCAATCCTTG-3′, and reverse, 5′-CACAAATTCCCATCATTCCC-3′; PIKCI: forward, 5′-TACGCGCAAGGAACCTCAAG-3′, and reverse, 5′-CTCGCAACTTGGTCACGTCT-3′. The primer sequences for GAPDH and β-actin were the following: GAPDH forward primer, 5′CGCTCTCTGCTCCTCCTGTTC-3′, GAPDH reverse primer, 5′-ATCCGTTGACTCCGACCTTCAC-3′; β-actin forward primer, 5′-CGCTCTCTGCTCCTCCTGTTC-3′, β-actin reverse primer, 5′-ATCCGTTGACTCCGACCTTC AC-3′. The U6 forward primer was 5′-CTCGCTTCGGCAGCACA-3′, and the U6 reverse primer was 5′-AACGCTTCACGAATTTGCGT-3′. The qRT-PCR reaction included an initial denaturation step at 95 °C for 10 min, which was then followed by 40 cycles of 92 °C for 15 s and 60 °C for 1 min. We used the ΔΔCt method to determine expression fold changes (tumor vs. normal) in subsequent calculations^30^.

### Nucleic Acid Electrophoresis

The cDNA and gDNA PCR products were investigated using 2% agarose gels with TE running buffer. The DNA was separated by electrophoresis at 110 V for 30 min. The DNA marker was DL600 (KeyGen, Nanjing, cat 37061). The results were examined by UV irradiation^31^.

### RNA isolation of nuclear and cytoplasmic fractions

The subcellular localization of hsa_circ_0067934 was detected using the PARIS™ Kit according to the manufacturer’s protocol (Ambion, Life Technologies, USA).

### Cell culture and siRNA transfection

TE-13 and KYSE-410 cells were donated by Professor Xinchen Sun (Department of Radiology, the First Affiliated Hospital of Nanjing Medical University). The ECA-109 and TE-1 cells were purchased from Shanghai Institutes for Biological Science, China. A normal human esophageal epithelial cell line (HEEC) was purchased from ScienCell Research Laboratories.

KYSE-410 and HEEC cells were grown in RPMI 1640 medium (KeyGen, Nanjing, China). The ECA-109, TE-1, and TE-13 cells were grown in DMEM (KeyGen, Nanjing, China), supplemented with penicillin-streptomycin and 10% FBS (Life Technologies, Australia). All cells were grown in at 37 °C in a humidified 5% CO_2_ atmosphere.

The ESCC cells were seeded at six-well plates and then transfected at 24 h with specific siRNA (100 NM) or control siRNA (100 NM) using Lipofectamine RNAi MAX according to the manufacturer’s protocol (Invitrogen). We used the following siRNA sequences: siRNA-1 for hsa_circ_0067934 sense: 5′UGUUGAUUGGGAUAUGUUAUU-3′; antisense: 5′UAACAUAUCCCAAUCAACAUU-3′; and siRNA2 for hsa_circ_0067934 sense: 5′-CCGAAAUGUUGAUUGGGAUTT-3′; antisense: 5′-AUCCCAAUCAACAUUUCGGTT-3′.

### Cell proliferation assay

Cell proliferation was assayed using the Cell Counting Kit-8 (CCK8) assay (Promega) according to the manufacturer’s protocol. The transfected cells were plated in 96-well plates (3000 cells/well). Cell proliferation was detected every 24 h according to the manufacturer’s protocol. Briefly, 10 μl of CCK 8 solution was added to each well and incubated for 2 h at 37 °C. The solution was then measured spectrophotometrically at 450 nm^32^.

### Clonogenic assay

We plated 100 transfected cells in a fresh six-well plate with 2 ml media containing 10% FBS. The medium was replaced every 3 days. The cells were immobilized with 4% paraformaldehyde and stained with 0.1% crystal violet two weeks later. The visible colonies were manually counted^33^.

### *In vitro* cell migration and invasion assays

We harvested TE-13 or ECA-109 cells transfected for 24 hours with 100-nM si-hsa_circ_0067934 or si-NC. The transfected cells (1 × 10^4^) were plated in the upper chamber of transwell assay inserts (Millipore, Billerica, MA, USA) containing 200 μl of serum-free DMEM with a membrane (8 mm pores) to test migration. The lower chambers were filled with RPMI1640 containing 10% FBS. The cells on the filter surface were fixed with methanol, stained with crystal violet, and photographed with a digital microscope after 24 h. The cell numbers were calculated in five random fields for each chamber.

The transfected cells (5 × 10^5^) were plated in the top chamber containing a Matrigel-coated membrane (BD Biosciences) in 500-μl serum-free DMEM to test cell invasion. There was 750 μl of 10% FBS-DMEM in the bottom chambers. The invasion function was determined after 48 h.

### Flow cytometric analysis

We harvested transfected cells after 24 h. The TE-13 and KYSE-410 cells were stained with Annexin V and PI using an Annexin V-FITC/PI apoptosis detection kit (BD Biosciences) before analysis by flow cytometry (FACScan; BD Biosciences). The cell cycle analysis was performed by staining cells with propidium iodide using the Cycle TEST PLUS DNA Reagent Kit (BD Biosciences). The cells were then analyzed using a FACScan analyzer^34^.

### Fluorescence *in situ* hybridization (FISH)

FISH was performed to detect subcellular location of hsa_circ_0067934 according to the method described by Vautrot V (pmid = 25791592). And FISH probe was designed to detect the splicing junction of two exons and the probe sequences was GTATGCGAATTTGTTTTT CCAAAATAACATATCCCAATCA.

### Western blot assay

The cellular proteins were extracted either 48 h after transfection or plating into a 6-well plate. The cells in each well were lysed in 100 μl of lysis buffer containing RIPA (99 μl) and protease inhibitor cocktail (1 μl). Then, 60 micrograms of protein were resolved by SDS–PAGE analysis and transferred onto a PVDF membrane. The primary antibodies used were anti-cyclin D (1:1000; CST) and anti-β-actin (1:1000; Abcam). The membranes were washed and then incubated with goat anti-rabbit HRP-conjugated secondary antibody (1: 10,000; Abcam) for 2 h at room temperature. The blots were visualized by ECL detection (Thermos Scientific)^35^.

### Statistical analysis

The Student’s t test and one-way ANOVA test or the nonparametric Kruskal-Wallis test, respectively was applied to assess the relationship between has_circ_0067934 expression and other characteristics. The strengths of the associations between continuous variables were tested with the Spearman correlation. The SPSS software package (version 20.0, SPSS Inc) was used to perform statistical analyses. A *P* < 0.05 was considered statistically significant.

## Additional Information

**How to cite this article**: Xia, W. *et al*. Circular RNA has_circ_0067934 is upregulated in esophageal squamous cell carcinoma and promoted proliferation. *Sci. Rep.* **6**, 35576; doi: 10.1038/srep35576 (2016).

## Supplementary Material

Supplementary Information

## Figures and Tables

**Table 1:** Correlation between has_circ_0067934 expression and clinic pathologic characteristics.

Characteristics	Number of patients	Fold change (mean)	P values
Age (years)			
<60	16	24.97	0.738
>60	35	26.47	
Gender			
Male	41	25.74	0.803
female	10	27.05	
Smoke			
Yes	21	27.79	0.473
No	30	24.75	
Drinking history			
No	40	26.08	0.945
Yes	11	25.73	
Family history			
Yes	4	23.05	0.726
No	47	26.21	
Cancer location			
Upper	9	25.28	0.916
Middle	30	26.72	
lower	12	24.74	
Tumor size			
<4 cm	31	27.61	0.335
>4 cm	20	23.05	
Tumor differentiation			
poorly	30	28.4	0.025*
High	14	17.64	
T stage			
I-II	14	23.26	0.04*
III-IV	37	32.96	
Lymph node metastasis			
N0-N1	18	24.53	0.601
N2-N3	33	26.8	
TNM stage			
I-II	22	20.48	0.021*
III-IV	29	30.19	

^*^Significant association.
