# Supplementary material for: Circular RNA has_circ_0067934 is upregulated in esophageal squamous cell carcinoma and promoted proliferation
Source: Sci Rep. 2016 Oct 18;6:35576. doi: 10.1038/srep35576 (PMC5067712; doi:10.1038/srep35576)
Supplement: Supplementary Information [file srep35576-s1.pdf]

Circular RNA has_circ_0067934 is upregulated in esophageal squamous cell carcinoma and promoted proliferation

Wenjia Xia , Mantang Qiu, Rui Chen, Siwei Wang, Xuechun Leng, Jie Wang， Youtao Xu,Jingwen Hu，Gaochao Dong，Lin Xu and Rong Yin.

Supplementary figure 1:

The figure was provided to indicate the site of splice junction, PCR primers, and FISH probes.

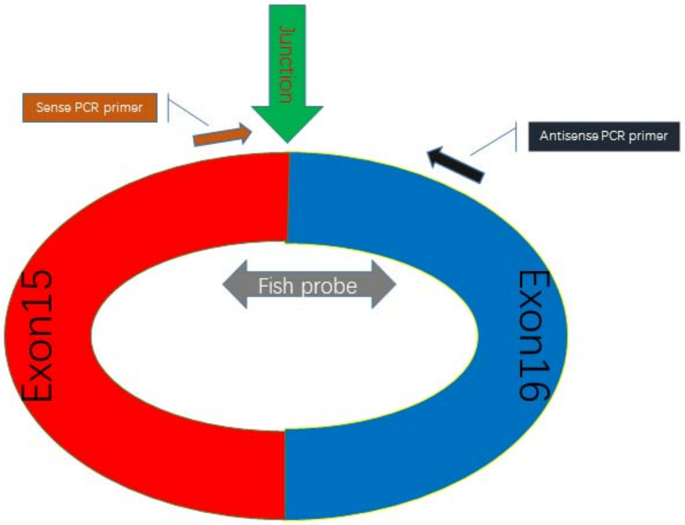

Supplementary figure 2:

The expression of hsa_circ_0067934 and PRKCI by qRT-PCR in a cohort of 26 samples, and the results showed that there was no significant correlation between hsa_circ_0067934 and PRKCI

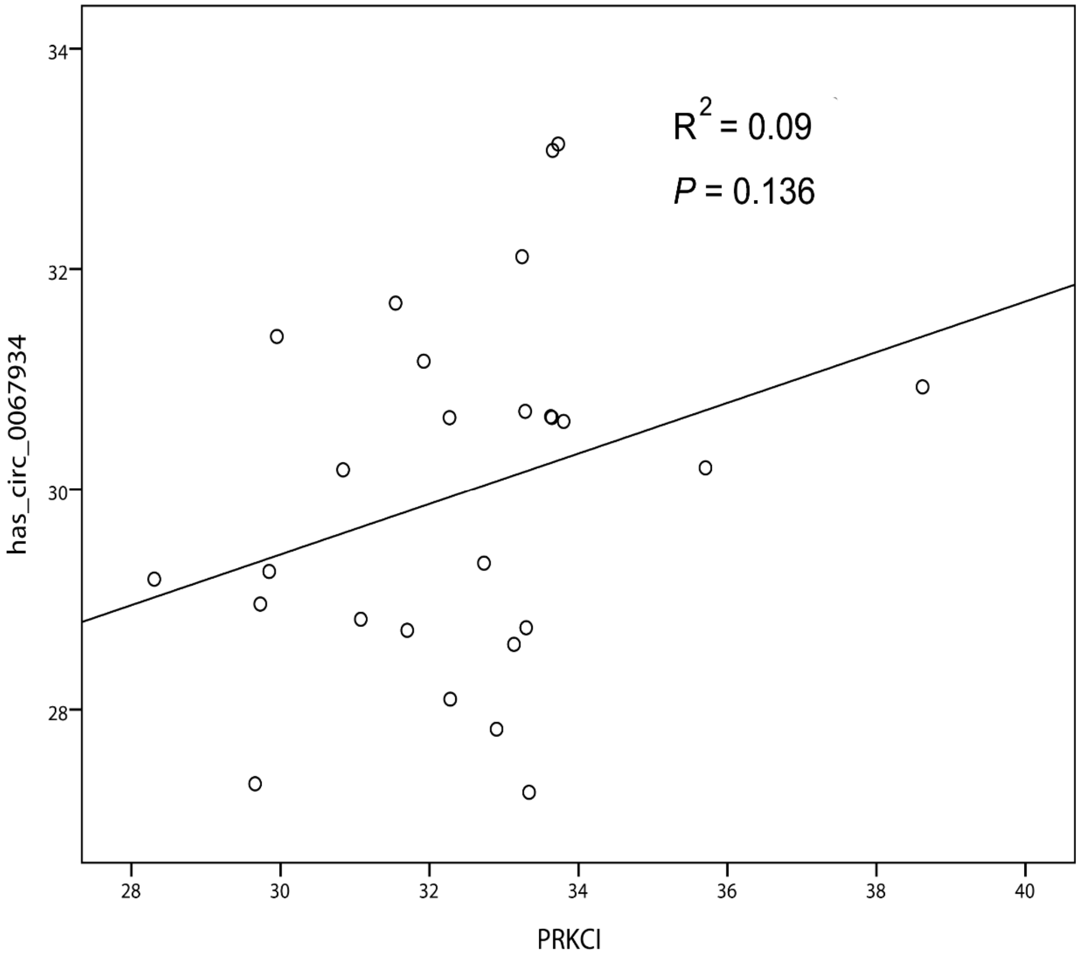

Supplementary file 3:

Flow cytometric analyses indicate that si-hsa_circ_0067934 did not affect cells apoptosis.

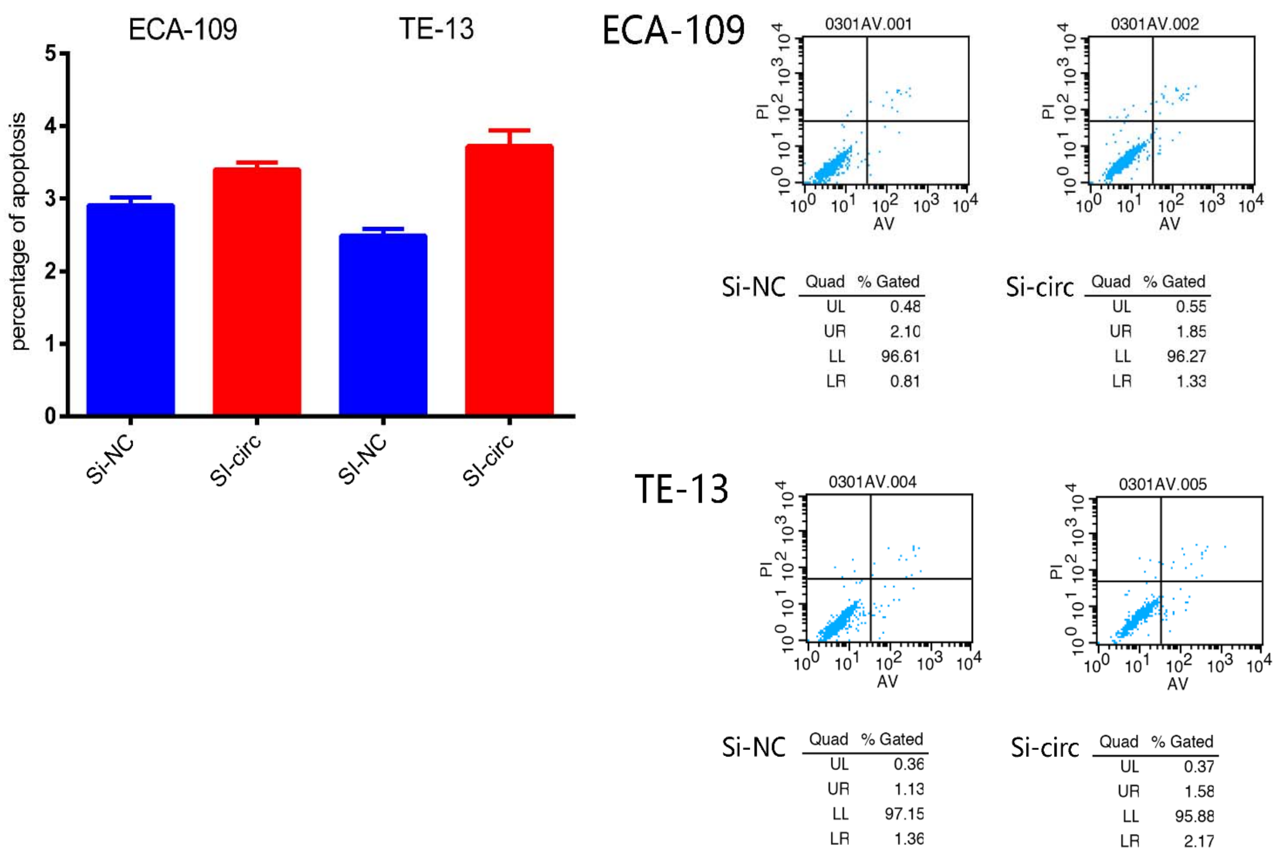

Supplementary file for review only：

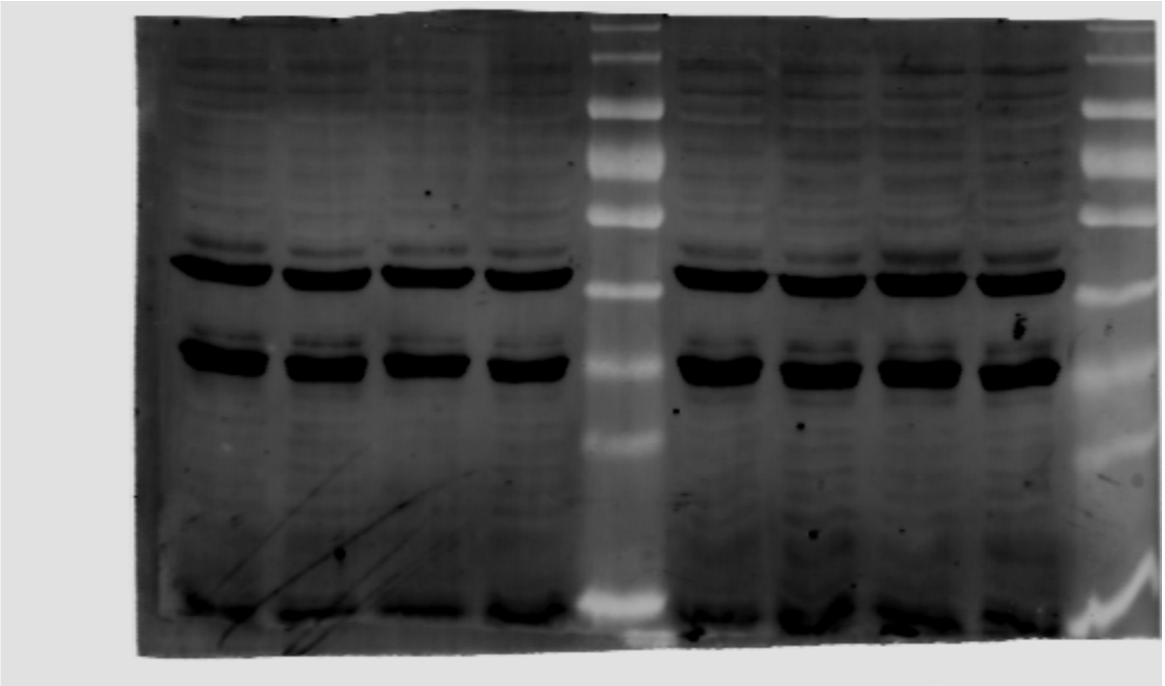

β-actin

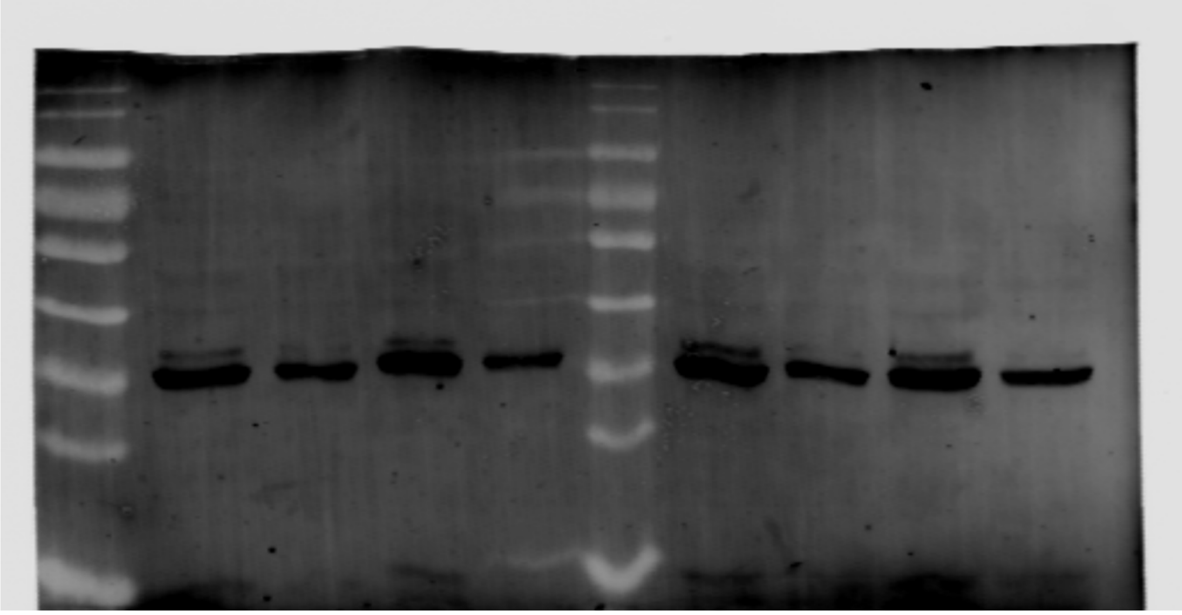

cyclinD
